# Unknown QRS Morphology Change at Peak Exercise: To Stop or to Continue?

**DOI:** 10.1016/j.jaccas.2023.101951

**Published:** 2023-08-22

**Authors:** Fabio M. Leonelli, Daniel Sun, Stephanie Gonzalez, Rejoy Sabin Thomas, Maqsood A. Siddique

**Affiliations:** a James A. Haley Veterans’ Hospital, Tampa, Florida, USA; b Tampa Veterans Affairs Clinical Research and Education Center, Tampa, Florida, USA

**Keywords:** exercise stress test, left septal fascicle block, septal depolarization

## Abstract

Electrocardiogram changes during stress tests are well standardized and understood. We present and explain a reversible QRS morphology change at peak exercise previously unreported. (**Level of Difficulty: Intermediate.**)

## Central Illustration

**Figure:**
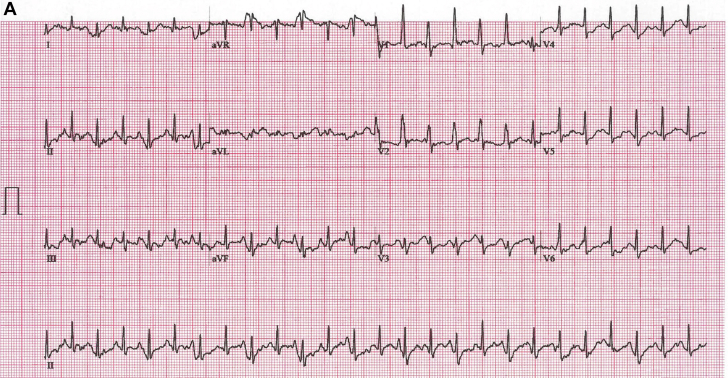

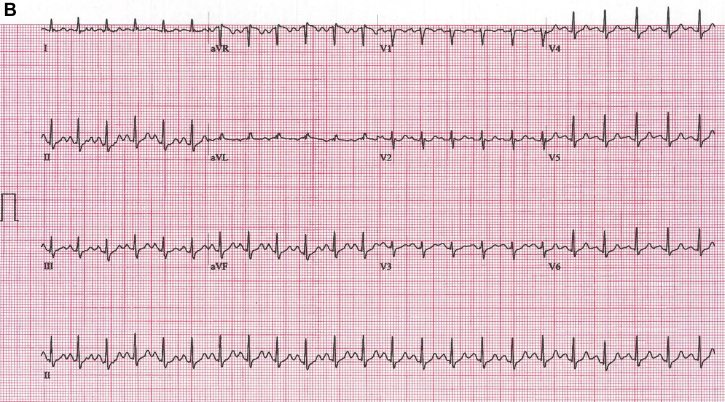

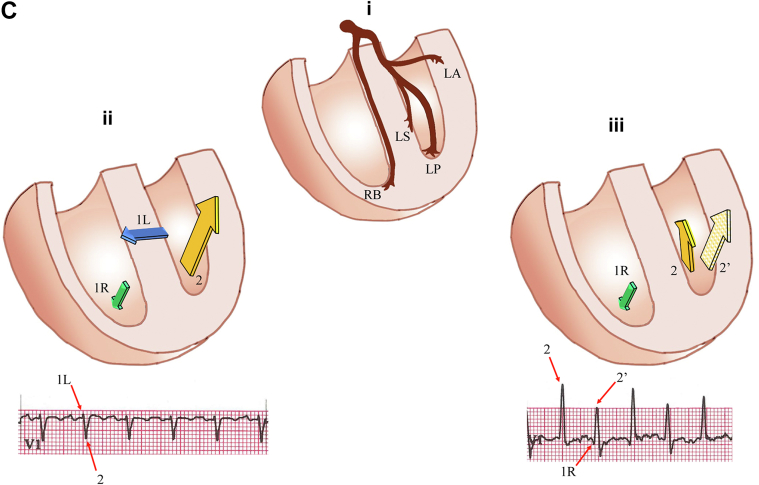

## Introduction

**Figure 1:**
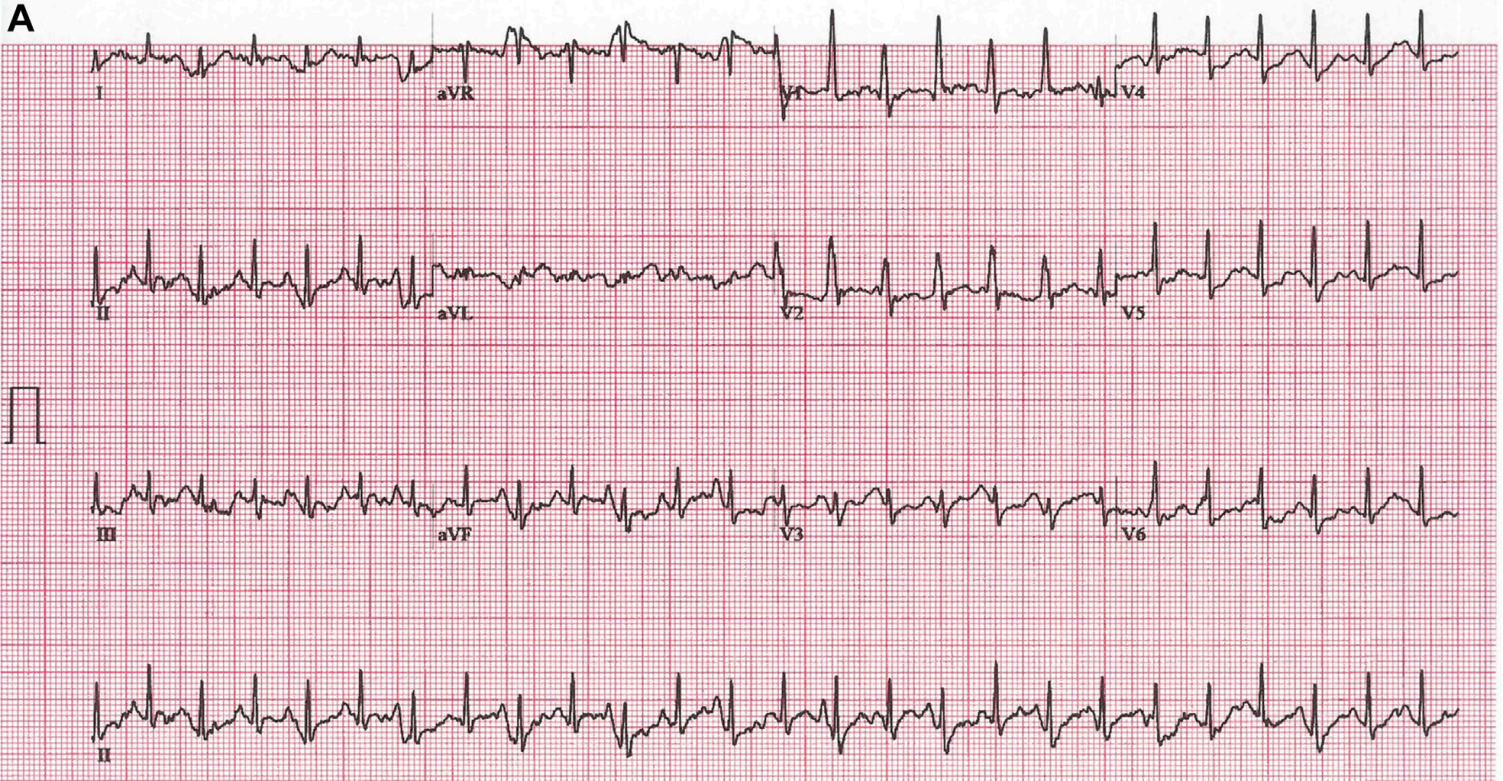

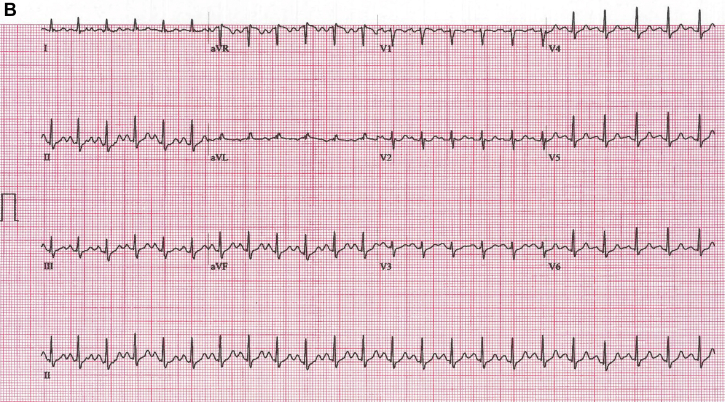

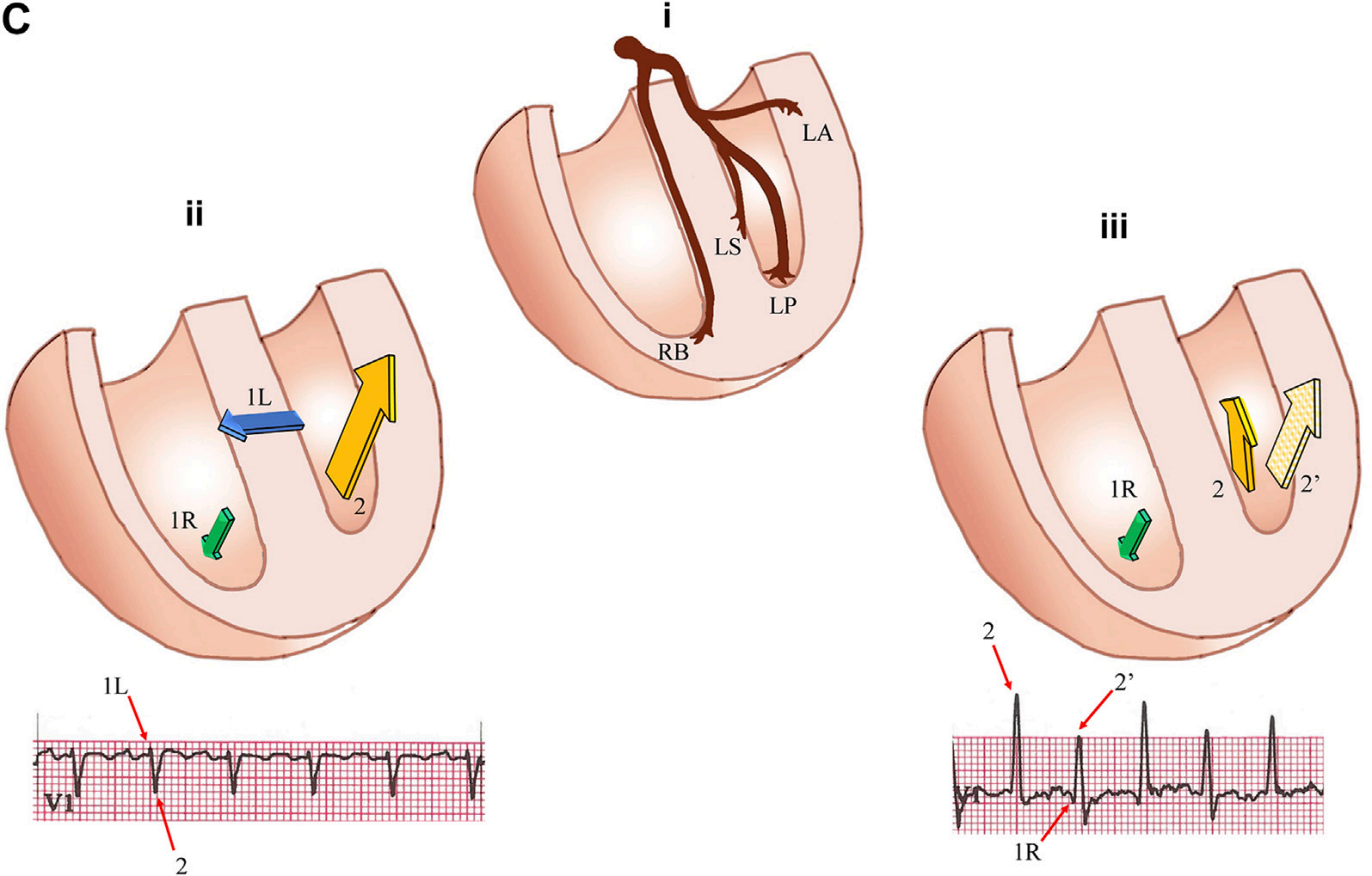
QRS Morphology at Peak Exercise and Recovery **(A)** Electrocardiogram (ECG) at peak exercise. Sinus tachycardia with a rate of 155 beats/min and sudden asymptomatic shift of horizontal plane vector anteriorly with voltage and QRS morphology alternans. **(B)** Recovery ECG with a sinus rate of 142 beats/min and normalization of the tracing as the speed and incline of the treadmill are reduced. **(C) (i)** Schematic representation of quadrifascicular division of the His-Purkinje system, including right bundle (RB) and left bundle fascicles with septal (LS), posterior (LP), and anterior fascicles (LA). **(ii)** Main vectors of normal cardiac depolarization including septal (vector 1) with left (1L, **blue arrow**) and right (1R, **green arrow**) components. Depolarization begins on the midanterior left side of the septum and, because of timing and muscle volume, inscribes a dominant vector directed anteriorly and left to right. Vector 2 is slightly delayed and, from the posteroseptal region, proceeds anterolaterally, enveloping the entire left ventricle (LV). In V_1_, both vectors are evident because of their divergent directions. **(iii)** Vectors observed in the case of delayed or blocked conduction along the septal branch (vector not represented). The R component (1R) of septal depolarization is unchanged, whereas the L septum is activated with some delay from the posteroseptal insertion of the left posterior fascicle (vector 2, **yellow arrow**). Because of this, LV activation is shifted more anteriorly, resulting in a dominant positive vector in V_1_. Delay of the septal branch may vary, at times creating, as in our case, an alternating pattern of conduction. Increasing delay of conduction in the L septal branch can make the R side vector (1R) dominant, inscribing a q in V_1_, and improved propagation will shift the overall LV vector toward the left with an R/S pattern in V_1_ (**checkered yellow arrow 2′**).

A 48-year-old woman was referred for a treadmill test to evaluate episodes of atypical chest pain of 6 weeks’ duration. Her functional capacity, cardiovascular examination, and echocardiographic left ventricular function findings were within normal limits. Baseline electrocardiogram (ECG) results were normal (Supplemental Figure 1). At stage 4 of the Bruce protocol, the ECG changed (Figure 1A). Despite a lack of symptoms, the test was stopped with rapid normalization of the changes (Figure 1B). Electrode displacement was excluded, and the patient recovered by decreasing the treadmill speed and incline.

## The changes at peak stress are most likely caused by which of the following?

A. Rate-related right bundle branch block
B. Right ventricular hypertrophy
C. Acute posterior myocardial infarction
D. Rate-related left septal fascicular block
E. Pre-excitation caused by the left lateral accessory pathway

## Discussion/Rationale

The baseline ECG results are normal. At peak exercise with a sinus rate of 155 beats/min, there is a sudden shift in the precordial vector with prominent anterior forces (V_1_-V_2_) without axis deviation or ST-T changes. V_1_ shows the R/S waveform voltage and q-wave alternans. The ECG normalizes during recovery as the sinus rate decreases to 142 beats/min.

Options A, B, and C can all cause prominent anterior forces, but option A is improbable with a normal QRS duration and absence of late conduction delay in the precordial leads. Rapid QRS normalization; absence of P, ST-T changes; and right axis deviation make option B unlikely. Option C is implausible in the absence of symptoms, repolarization abnormalities, and rapid ECG normalization. Absence of a delta wave, unchanged PR, and R-wave in I and aVL make option E unlikely. The correct answer is D.

The function and clinical significance of the left septal fascicle (LSF) is more controversial than the other 2 branches of the left bundle despite anatomic studies describing its presence and variable extension.^1^

Activation of the interventricular septum (IVS) begins on the left side, rapidly followed by depolarization of the right septal surface (Figure 1Ci) carried by the right bundle (Figure 1Cii). Delayed activation of the left IVS results in variable QRS changes depending on the dimension and arborization of the LSF and the severity of the conduction delay of this fascicle.

In cases of minimal delay, normally conducting right septal branches will become dominant, generating an R-to-L posteriorly directed vector with q waves in the anterior leads and the disappearance of r waves in V_4_ to V_6_.

Greater conduction delays of a larger LSF will make the entire left IVS activation dependent on activation from the left posterior fascicle shifting the left ventricular vector in a back-to-front, left-to-right direction (Figure 1Ciii), generating, in the horizontal plane, a QRS loop initially directed posteriorly and, later, anteriorly and leftward. The QRS duration and frontal plain axis will be unchanged because the depolarization of the majority of the left ventricle is unaffected. Based on this model, ECG criteria of left septal fascicle block (LSFB)^2^ have been proposed and confirmed by numerous clinical observations.

Our patient, at a rate of 154 beats/min, suddenly shifts the depolarization vector anteriorly with alternans of voltage and morphology (Figure 1A). QRS alternates between a dominant R and lower-voltage R/S, most likely because of variable delay in the LSFB. The former represents a complete LSFB, whereas the latter shows a partial recovery of the LSFB with a more posterior shift of the overall left ventricular vector.^3^ The presence of alternating q waves with the R is also in keeping with different degrees of LSFB delay.

The absence of repolarization changes and symptoms of angina strongly supports the hypothesis of a rate-related LSFB. The stress test could have been continued because the septal block would not have interfered with recognition of ischemic changes.

## Funding Support and Author Disclosures

The authors have reported that they have no relationships relevant to the contents of this paper to disclose.
