# Supplementary figures and images for: Unknown QRS Morphology Change at Peak Exercise: To Stop or to Continue?
Source: JACC Case Rep. 2023 Aug 22;22:101951. doi: 10.1016/j.jaccas.2023.101951 (PMC10544094; doi:10.1016/j.jaccas.2023.101951)

**Supplemental Figure 1.** **Baseline ECG prior to exercise stress test. Showing a normal tracing.**


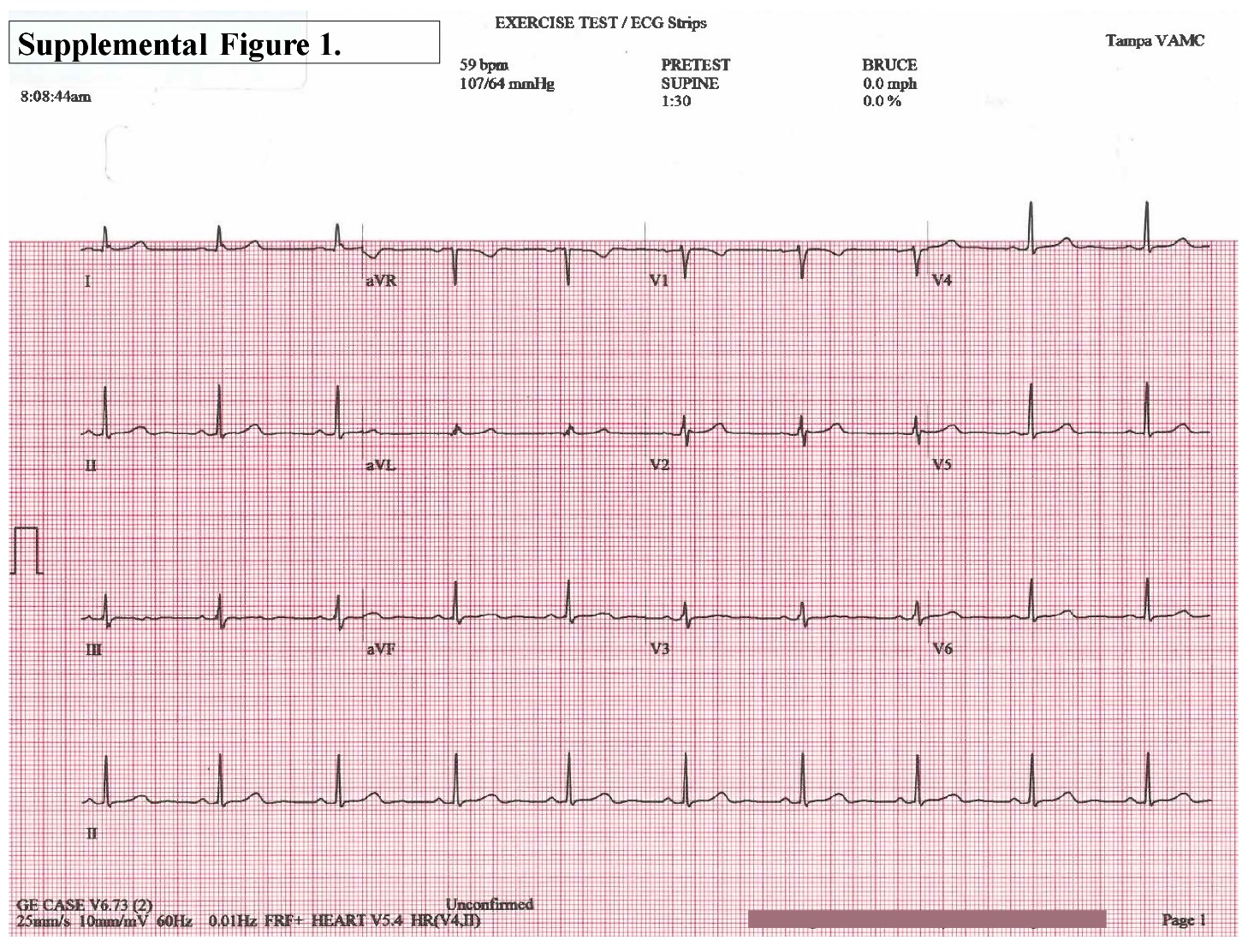

Supplement: Supplemental Figure 1 [file mmc1.docx]
